# Clinical effects of acupuncture for traumatic peripheral nerve injury: a meta-analysis of randomized controlled trials

**DOI:** 10.3389/fneur.2026.1852669

**Published:** 2026-10-16

**Authors:** Wanying Jia, Ruonan Liang, Yuqi Duan, Yidan Xu, Xinning Ran, Yingdong Wang, Chenduo Li, Yi Guo, Mingxing Zhang

**Affiliations:** 1 School of Integrative Medicine, Tianjin University of Traditional Chinese Medicine, Tianjin, China; 2 School of Acupuncture & Moxibustion and Tuina, Tianjin University of Traditional Chinese Medicine, Tianjin, China; 3 Research Center of Experimental Acupuncture Science, Tianjin University of Traditional Chinese Medicine, Tianjin, China

**Keywords:** acupuncture, meta-analysis, nerve plexus, randomized controlled trial, traumatic peripheral nerve injury

## Abstract

**Objective:**

This study aimed to systematically evaluate the clinical efficacy of acupuncture in the treatment of traumatic peripheral nerve injury (TPNI), and to provide more reliable evidence for high-quality clinical practice and decision-making.

**Methods:**

Randomized controlled trials (RCTs) on acupuncture for TPNI were retrieved from China National Knowledge Infrastructure (CNKI), Chongqing VIP Information Co., Ltd. (VIP), Wan Fang, China Biomedical Literature Database, PubMed, Cochrane Library, Web of Science, and Embase from database inception to October 2025. Two researchers independently screened the literature according to inclusion and exclusion criteria, extracted data, and assessed the risk of bias. Meta-analysis was performed using Review Manager 5.4 software.

**Results:**

A total of 36 RCTs involving 2,798 patients were included. Meta-analysis showed that, compared with the non-acupuncture control group, acupuncture improved the total clinical effective rate (RR = 1.24, 95% CI [1.20, 1.28], *p* < 0.00001, I^2^ = 27%), promoted neurological function score (MD = 1.04, 95% CI [0.53, 1.54], *p* < 0.0001, I^2^ = 79%), reduced distal motor latency (DML) (MD = −0.49, 95% CI [−0.78, −0.20], *p* = 0.0008, I^2^ = 79%), increased compound muscle action potential amplitude (CMAP) (MD = 1.88, 95% CI [0.20, 3.56], *p* = 0.03, I^2^ = 98%), and raised sensory nerve conduction velocity (SCV) (MD = 5.14, 95% CI [2.33, 7.95], *p* = 0.003, I^2^ = 81%). However, trial sequential analysis indicated that the cumulative sample sizes for CMAP and SCV did not reach the required information size, suggesting that the stability of the above findings remains limited. There was no significant difference in clinical effective rate and neurological function score among patients with different nerve plexus injuries. The improvement of DML, CMAP, and SCV in patients with brachial plexus injury was better than that in patients with lumbosacral plexus injury. The overall certainty of evidence in this study was rated as low.

**Conclusion:**

Acupuncture can significantly improve the clinical effectiveness of traumatic peripheral nerve injury and is associated with no adverse reactions. However, given the suboptimal methodological quality of the included reviews, further well-designed randomized controlled trials are warranted to confirm our findings.

**Systematic review registration:**

https://www.crd.york.ac.uk/PROSPERO/view/CRD420251168215, CRD420251168215.

## 1 Introduction

Traumatic peripheral nerve injury (TPNI) is one of the most common diseases in clinical practice, mainly manifested as sensory disturbances in the area innervated by the injured nerve, limb paralysis and atrophy, and neuropathic pain (1). According to statistics, in developed countries, the annual incidence of TPNI is approximately 13 to 23 cases per 100,000 people (2). At the same time, this type of injury accounts for 1.5 to 4.0% of newly reported trauma cases worldwide each year (3). Although peripheral nerves have a certain capacity for regeneration, due to the slow regeneration process and complex structure, target organs and tissues cannot be reinnervated in time, resulting in irreversible damage (4). Conventional surgical treatments often fail to achieve ideal recovery of nerve function. Therefore, exploring effective adjunctive therapies has become an urgent need in current clinical treatment.

Acupuncture, as an ancient therapy originating from traditional Chinese medicine, has a history of more than 3,000 years and is gradually gaining global attention (5). Experimental studies have shown that acupuncture may promote the recovery of nerve function by increasing the expression of neurotrophic factors, inhibiting neuroinflammation, and promoting angiogenesis (6–8). Clinical practice has also shown that acupuncture can improve functional recovery after peripheral nerve injury; however, there is a lack of systematic analysis of high-quality randomized controlled trials and overall evaluation of its efficacy.

Therefore, this study integrated randomized controlled trials of acupuncture in the treatment of peripheral nerve injury. Clinical effective rate, neurological function score, distal motor latency (DML), compound muscle action potential amplitude (CMAP), and sensory nerve conduction velocity (SCV) were used as outcome indicators. Subgroup analysis was performed based on nerve plexus to systematically evaluate the clinical efficacy of acupuncture for TPNI and to provide more reliable evidence for high-quality clinical practice and decision-making.

## 2 Materials and methods

This systematic review was registered on the PROSPERO database (Registration number: CRD420251168215). The review was conducted in accordance with the Preferred Reporting Items for Systematic reviews and Meta-Analyses (PRISMA) 2020 statement and the Cochrane Handbook for Systematic Reviews of Interventions.

### 2.1 Search strategy and selection

Eight databases, including China National Knowledge Infrastructure (CNKI), *Chongqing VIP Information Co., Ltd.* (VIP), Wan Fang, China Biomedical Literature Database, PubMed, Cochrane Library, Web of Science, and Embase, were independently searched by two authors. The search period was from database inception to October 17, 2025. In addition, relevant journals and reference lists of included studies were manually searched to supplement eligible studies.

A combination of subject terms and free-text terms was used for retrieval. The search process is shown in Table 1. The study selection process was as follows: first, search for acupuncture, peripheral nerve, injury, and randomized controlled trials in each database; second, remove duplicate records; third, screen titles and abstracts; fourth, conduct full-text review; fifth, supplement eligible studies through reference lists; sixth, merge the included studies from both methods.

**Table 1:** Presents the PRISMA flow diagram of the study selection process.

Search and selection strategy	
#1	Acupuncture(mesh)
#2	acupuncture therapy
#3	pharmacopuncture
#4	acupuncture treatment
#5	acupotomy
#6	ear acupuncture
#7	auricular acupuncture
#8	electroacupuncture
#9	electro-acupuncture
#10	fire acupuncture
#11	transcutaneous electrical acupoint stimulation
#12	TEAS
#13	scalp needle
#14	scalp acupuncture
#15	abdominal needle
#16	abdominal acupuncture
#17	warm needle
#18	warm acupuncture
#19	triangular needle
#20	triangular acupuncture
#21	#1 or #2 or #3 or #4 or #5 or #6 or #7 or #8 or #9 or #10 or #11 or #12 or #13 or #14 or #15 or #16 or #17 or #18 or #19 or #20
#22	peripheral nerve(mesh)
#23	endoneurium
#24	epineurium
#25	perineurium
#26	PN
#27	brachial plexus
#28	musculocutaneous nerve
#29	median nerve
#30	ulnar nerve
#31	radial nerve
#32	axillary nerve
#33	femoral nerve
#34	sciatic nerve
#35	nervi ischiadicus
#36	tibial nerve
#37	nervus peroneus communis
#38	lumbar plexus
#39	sacral plexus
#40	#22 or #23 or #24 or #25 or #26 or #27 or #28 or #29 or #30 or #31 or #32 or #33 or #34 or #35 or #36 or #37 or #37 or #38 or #39
#41	injuries
#42	damnification
#43	detriment
#44	impairment
#45	impairment
#46	scathe
#47	bound
#48	trauma
#49	damage
#50	#41 or #42 or #43 or #44 or #45 or #46 or #47 or #48 or #49
#51	randomized controlled trial
#52	clinical trial
#53	controlled clinical trial
#54	RCT
#55	#51 or #52 or #53 or #54
#56	humans(mesh)
#57	#21 and #40 and #50 and #55 and #56

### 2.2 Inclusion criteria

(1) Study type: published randomized controlled trials on acupuncture for peripheral nerve injury, with articles in Chinese or English. (2) Participants: patients diagnosed with traumatic peripheral nerve injury, regardless of age, sex, or baseline disease severity. (3) Interventions: the experimental group received acupuncture, needling, electroacupuncture, or acupotomy (specific techniques, acupoints, and treatment duration were not limited), with or without the same measures as the control group. The control group received non-acupuncture therapy or sham acupuncture, such as medicine, rehabilitation training, physical therapy, and biofeedback. (4) The primary outcome measure was the clinical effective rate, which was required to be reported in all included studies. The secondary outcome measures included neurological function scores, DML, CMAP, and SCV. If a study reported one or more of these measures, the corresponding data were extracted for analysis; it was not required that all studies report all secondary outcomes.

### 2.3 Exclusion criteria

(1) Non-randomized controlled studies. (2) Animal experiments or studies not involving peripheral nerve injury. (3) Studies on nerves located in the trunk and head/face (excluded due to limitations in detection technique consistency). (4) Studies with acupuncture interventions in the control group. (5) Studies with unavailable full text or unclear/incomplete outcome data. (6) Review articles, dissertations, case reports, or experience reports. (7) Duplicate publications (the earliest or most complete study was included).

### 2.4 Study selection and data extraction

Two researchers independently conducted study selection and data extraction. The extracted data included first author, publication year, country, language, sample size, characteristics of participants (age, sex, disease type), interventions, control measures, treatment duration, and outcome indicators. If, A third party would be consulted, when there were disagreements between the two researchers. If The original authors would be contacted, when the information was insufficient.

### 2.5 Statistical analysis

RevMan 5.4 software was used for meta-analysis. Heterogeneity was evaluated using the I^2^ statistic. If I^2^ ≥ 50% and *p* < 0.10, significant heterogeneity was considered present, and a random-effects model was applied; otherwise, a fixed-effects model was used. When more than 10 RCTs were included for an outcome, Egger’s test was performed using Stata 17.0 to assess publication bias. A two-sided *p* < 0.05 was considered statistically significant. Trial sequential analysis (TSA 0.9.5.10; Copenhagen Trial Unit, Denmark) was conducted for primary outcomes to calculate the required information size (RIS). Type I error was set at α = 0.05, the type II error at β = 0.20 (power = 80%), in order to reduce random error and evaluate the robustness of the pooled results.

### 2.6 Certainty of evidence

The certainty of evidence was assessed using the Grading of Recommendations, Assessment, Development and Evaluations (GRADE) system, and each outcome was classified into one of four categories: high, moderate, low, and very low. High certainty indicates that the true effect is very close to the estimated effect; moderate certainty suggests that the true effect is likely close to the estimated effect but there is a possibility of substantial difference; low certainty implies that the true effect may be substantially different from the estimated effect; and very low certainty indicates that the true effect is probably markedly different from the estimated effect.

## 3 Results

### 3.1 Study selection

A total of 1,088 records were initially retrieved. After removing 300 duplicate records, 717 studies were excluded through title and abstract screening. Two studies were excluded due to unavailable full text, and 37 studies were excluded after full-text review. Five additional studies were included through reference screening, and one study was excluded after full-text assessment. Finally, 36 studies (9–44) meeting the criteria were included.

All included studies were published in Chinese, and no English studies were included. The total number of participants in the included studies was 2,798. The study selection process is shown in Figure 1, and the basic characteristics of the included studies are shown in Table 2.

**Figure 1:**
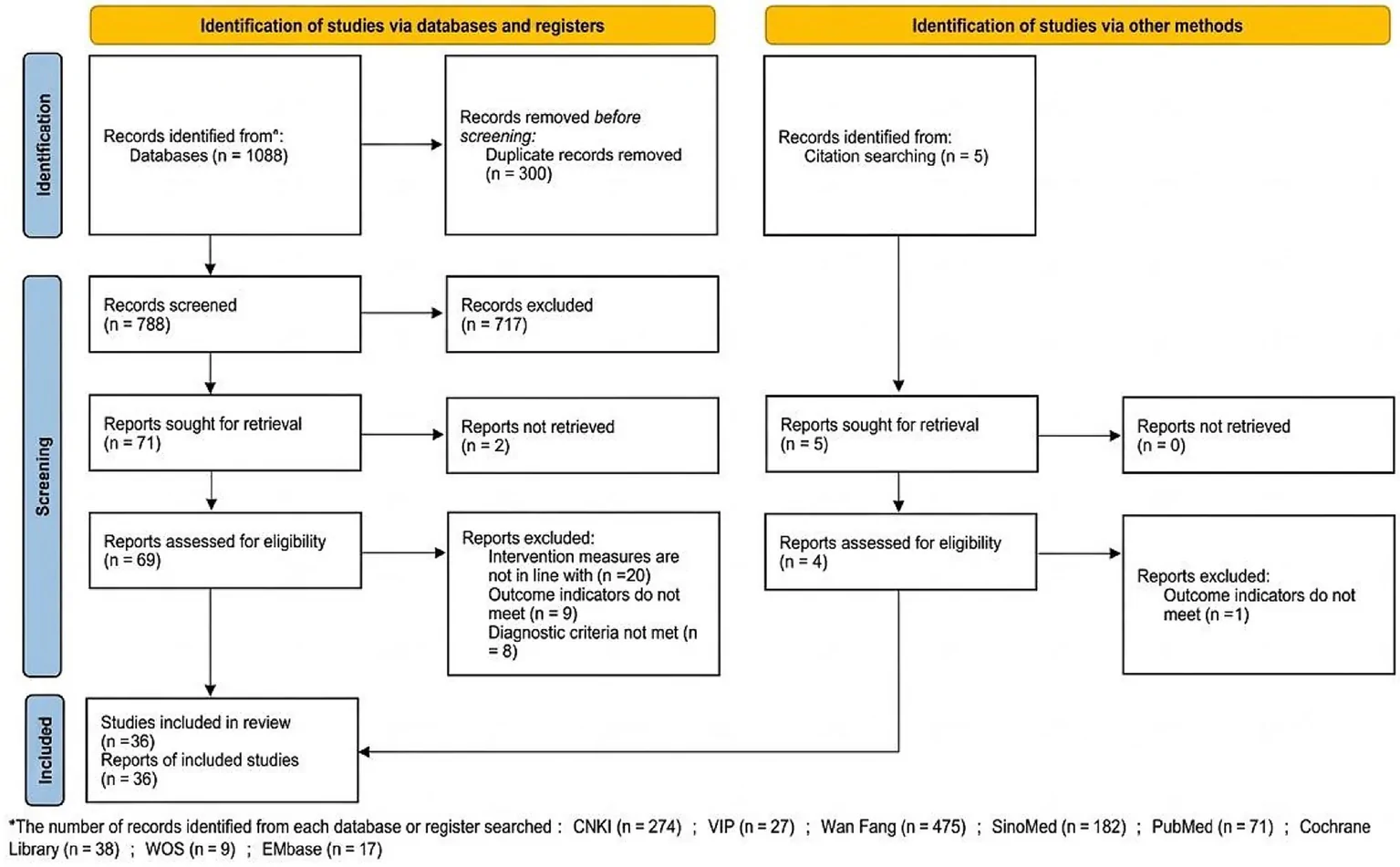
Literature screening flow diagram.

**Table 2:** Basic characteristics of included studies.

Serial number	Author/year	Injury nerve	Sample size	Gender (male/female)	Age (years)	Intervention	Intervention time	Outcome indicators
1	Yu (42)	Upper limb peripheral nerves	Ex:15	Ex:8/7	Ex:42.8 ± 3.4	Ex:acupuncture+drug	30d	Clinical efficacy,neurological function score
			Co:15	Co:9/6	Co:41.6 ± 3.7	Co:drug		
2	Ye et al. (44)	Upper limb peripheral nerves	Ex:15	Ex:9/6	Ex:42.22 ± 6.22	Ex: EA + PNF and conventional	2 m	Clinical efficacy
			Co:15	Co:9/6	Co:42.10 ± 6.16	Co: PNF and conventional		
3	Pang et al. (23)	Upper limb peripheral nerves	Ex:47	Ex:31/16	Ex:34.32–6.51	Ex: EA + rehabilitation therapy	8w	Clinical efficacy, CMAP
			Co:47	Co:29/18	Co:34.12 ± 6.47	Co:rehabilitation therapy		
4	Wang et al. (17)	Upper limb peripheral nerves	Ex:39	Ex:27/12	/	Ex: EA + rehabilitation therapy	60d	Clinical efficacy
			Co:33	Co:21/12	/	Co:rehabilitation therapy		
5	Zhao and Lin (22)	Upper limb peripheral nerves	Ex:29	/	/	Ex: EA + rehabilitation therapy	60d	Clinical efficacy
			Co:29	/	/	Co:rehabilitation therapy		
6	Guo (16)	Upper limb peripheral nerves	Ex:56	Ex:35/21	Ex:42.3 ± 7.8	Ex: EA + rehabilitation therapy	/	Clinical efficacy
			Co:56	Co:33/23	Co:40.2 ± 8.3	Co:rehabilitation therapy		
7	Qian et al. (27)	Upper limb peripheral nerves	Ex:45	Ex:32/13	Ex:48.6 ± 2.3	Ex:acupuncture+rehabilitation therapy	3 m	Clinical efficacy, CMAP
			Co:45	Co:33/12	Co:49.1 ± 2.2	Co:rehabilitation therapy		
8	Han et al. (33)	Upper limb peripheral nerves	Ex:30	/	/	Ex:acupuncture+drug	1 m	Clinical efficacy
			Co:30	/	/	Co:drug		
9	Meng et al. (18)	Brachial plexus	Ex:105	Ex:73/32	Ex:35.66 ± 2.47	Ex: EA + drug,rehabilitation therapy	3 m	Clinical efficacy
			Co:103	Co:72/31	Co:33.57 ± 2.19	Co:drug,rehabilitation therapy		
10	Du (38)	Brachial plexus	Ex:30	Ex:17/13	/	Ex:acupuncture+drug and exercise	56d	Clinical efficacy
			Co:30	Co:16/14	/	Co:drug and exercise		
11	Tang (25)	Brachial plexus	Ex:30	Ex:19/11	Ex:35.1 ± 4.0	Ex:warm acupuncture+drug	6w	Clinical efficacy,neurological function score
			Co:30	Co:30/10	Co:34.9 ± 3.8	Co:drug		
12	Xiong et al. (9)	Brachial plexus	Ex:40	Ex:22/18	Ex:1.1 ± 0.4	Ex: EA + rehabilitation therapy	4 m	Clinical efficacy,neurological function score
			Co:40	Co:25/15	Co:1.2 ± 0.5	Co:rehabilitation therapy		
13	Deng (11)	Brachial plexus	Ex:38	Ex:21/17	Ex:0.88	Ex: EA + rehabilitation therapy	60d	Clinical efficacy
			Co:33	Co:18/14	Co:0.89	Co:rehabilitation therapy		
14	Qiu et al. (36)	Brachial plexus	Ex:32	/	/	Ex:acupotomy+tuina	3 m	Clinical efficacy
			Co:31	/	/	Co:tuina		
15	Yan et al. (28)	Brachial plexus	Ex:43	Ex:25/18	Ex:0.88	Ex:acupuncture+exercise	90d	Clinical efficacy
			Co:25	Co:15/10	Co:0.88	Co:exercise		
16	Huang and Li (15)	Brachial plexus	Ex:43	Ex:32/11	Ex:34.98 ± 2.17	Ex: EA + conventional	1 m	Clinical efficacy,neurological function score
			Co:42	Co:35/7	Co:35.04 ± 2.32	Co:conventional		
17	Cong (35)	Median nerve	Ex:35	Ex:11/24	Ex:46.32 ± 5.57	Ex:acupotomy+rehabilitation therapy	21d	Clinical efficacy, DML, SCV
			Co:35	Co:12/23	Co:46.33 ± 5.55	Co:rehabilitation therapy		
18	Yang et al. (19)	Median nerve	Ex:37	Ex:26/11	Ex:32.45 ± 5.22	Ex: EA + exercise and drug	12w	Clinical efficacy, DML, CMAP, SCV
			Co:37	Co:23/14	Co:32.45 ± 5.22	Co:exercise and drug		
19	Hu and Luan (37)	Median nerve	Ex:20	Ex:7/13	/	Ex:acupotomy	21d	Clinical efficacy
			Co:20	Co:6/14	/	Co:block therapy		
20	Liu (32)	Radial nerve	Ex:23	Ex:15/8	/	Ex:acupuncture	40d	Clinical efficacy
			Co:22	Co:13/9	/	Co:drug		
21	Sun (10)	Radial nerve	Ex:52	Ex:32/20	Ex:41.16 ± 5.32	Ex: EA + drug	20d	Clinical efficacy, SCV
			Co:52	Co:30/22	Co:41.20 ± 5.27	Co:drug		
22	Zhang and Zhang (41)	Radial nerve	Ex:77	Ex:40/37	Ex:58.72 ± 2.80	Ex:warm acupuncture+drug,rehabilitation therapy and BFT	4w	Clinical efficacy
			Co:77	Co:39/38	Co:58.69 ± 2.78	Co:drug,rehabilitation therapy and BFT		
23	Li and Cai (12)	Radial nerve	Ex:52	Ex:32/20	Ex:28.82 ± 6.78	Ex: EA	30d	Clinical efficacy
			Co:53	Co:35/18	Co:29.78 ± 6.42	Co:rehabilitation therapy		
24	Sheng (39)	Radial nerve	Ex:28	Ex:19/9	Ex:32 ± 3.56	Ex:acupuncture	3 m	Clinical efficacy
			Co:28	Co:19/9	Co:30 ± 3.25	Co:drug		
25	Cai (31)	Radial nerve	Ex:30	Ex:21/9	Ex:36.83 ± 7.51	Ex:acupuncture	30d	Clinical efficacy
			Co:30	Co:22/8	Co:39.72 ± 8.64	Co:drug		
26	Deng et al. (26)	Radial nerve	Ex:20	/	/	Ex:warm acupuncture+drug	4w	Clinical efficacy
			Co:20	/	/	Co:drug		
27	Zou and Ye (21)	Radial nerve	Ex:40	/	/	Ex: EA	30d	Clinical efficacy
			Co:40	/	/	Co:nerve injury treatment instrument		
28	Zhong et al. (20)	Radial nerve	Ex:43	Ex:23/20	Ex:39.21 ± 10.35	Ex: EA + rehabilitation therapy	20d	Clinical efficacy
			Co:43	Co:21/22	Co:38.93 ± 10.46	Co:rehabilitation therapy and drug		
29	Ma and Sun (40)	Radial nerve	Ex:40	Ex:28/12	Ex:42.34 ± 7.41	Ex:acupuncture+drug	40d	Clinical efficacy, SCV
			Co:40	Co:26/14	Co:42.39 ± 7.35	Co:drug		
30	Guo et al. (34)	Ulnar nerve	Ex:20	Ex:8/12	Ex:54.15	Ex:acupuncture+drug	40d	Clinical efficacy
			Co:20	Co:9/11	Co:51.85	Co:drug		
31	Cai and Lv (43)	Sciatic nerve	Ex:31	Ex:18/13	Ex:14.2 ± 3.8	Ex: EA + drug	1 m	Clinical efficacy
			Co:31	Co:19/12	Co:13.7 ± 5.4	Co:drug		
32	Zhang et al. (30)	Nervus peroneus communis	Ex:30	Ex:20/10	/	Ex:acupuncture		
			Co:30	Co:19/11	/	Co: TCMF	20d	Clinical efficacy
33	Li et al. (29)	Nervus peroneus communis	Ex:65	Ex:37/28	/	Ex:acupuncture	30d	Clinical efficacy
			Co:32	Co:19/13	/	Co:drug		
34	Tu et al. (14)	Nervus peroneus communis	Ex:56	/	/	Ex: EA + rehabilitation therapy		
			Co:56	/	/	Co:rehabilitation therapy	4w	Clinical efficacy
35	He and Zhang (13)	Nervus peroneus communis	Ex:41	Ex:28/13	Ex:49.8 ± 4.9	Ex: EA + tuina		
			Co:41	Co:26/15	Co:48.7 ± 4.7	Co:tuina	4w	Clinical efficacy, DML, CMAP, SCV
36	Chen and Hou (24)	Nervus peroneus communis	Ex:55	Ex:30/25	Ex:64.68 ± 7.24	Ex:warm acupuncture+drug		
			Co:55	Co:27/28	Co:65.12 ± 7.33	Co:drug	12w	Clinical efficacy,neurological function score

EA, Electroacupuncture; BFT, Biofeedback training; TCMF, Traditional Chinese medicine fumigation.

### 3.2 Risk of bias assessment

Two researchers independently assessed the risk of bias of 36 included studies (9–44) according to the Cochrane risk of bias tool using RevMan 5.4 software. The assessment domains included random sequence generation, allocation concealment, blinding of participants and personnel, blinding of outcome assessment, completeness of outcome data, selective reporting, and other sources of bias. Among them, 13 studies (9, 10, 12–14, 19, 20, 23, 25, 27, 40, 41, 44) used a random number table for randomization, 1 study (36) used random number envelopes, 1 study (35) used case numbers for randomization, 4 studies (29–32) allocated participants according to the order of admission, 15 studies (15–18, 21, 22, 24, 26, 33, 37–39, 42, 43) only mentioned randomization without detailed description, and 2 studies (11, 28) allocated participants based on whether the parents of pediatric patients agreed to acupuncture treatment. Only 1 study (42) reported allocation concealment. Regarding blinding of participants and personnel, none of the 36 studies (9–44) described blinding in detail; considering that acupuncture procedures are difficult to blind for practitioners and no sham acupuncture was implemented, blinding was judged as not feasible. For blinding of outcome assessment, 2 studies (39, 42) reported blinding of outcome assessors, while the remaining studies did not describe this. Other potential sources of bias may exist. All included studies clearly reported the intervention protocols for both groups and the methods of data analysis.

The results of the risk of bias assessment are shown in Figures 2A,B.

**Figure 2:**
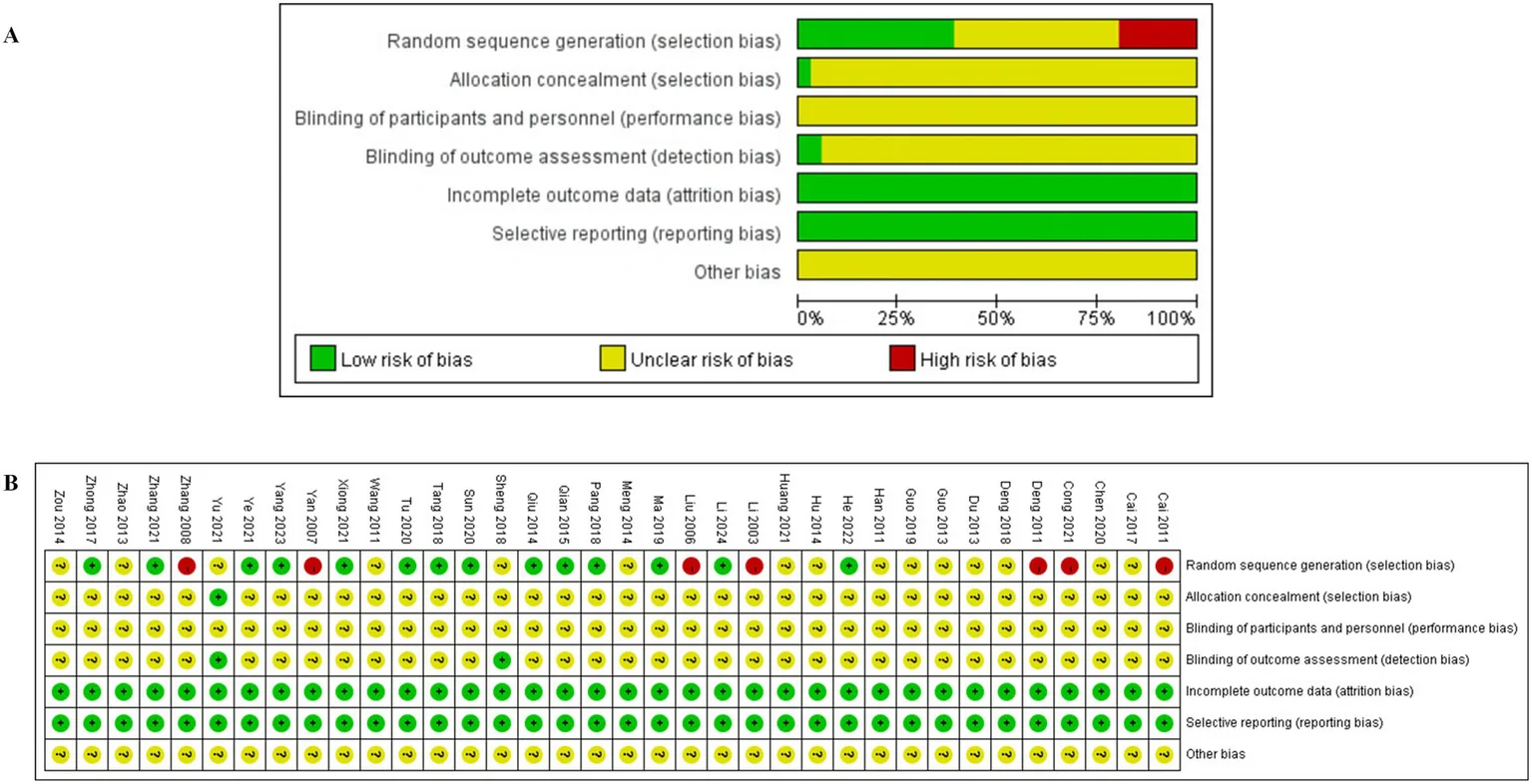
**(A)** Risk of bias graph. **(B)** Risk of bias summary.

### 3.3 Meta-analysis results

#### 3.3.1 Clinical effective rate

A total of 36 studies (9–44) reported the comparison of clinical effectiveness before and after treatment, with a total sample size of 2,798 cases. No heterogeneity was observed among the studies (I^2^ = 27%, *p* = 0.07), and a fixed-effects model was used for analysis. After acupuncture treatment, the clinical effectiveness of the affected nerves was significantly improved compared with the control group (RR = 1.24, 95% CI [1.20, 1.28], *p* < 0.00001; Figure 3A). Subgroup analysis based on nerve plexus showed that acupuncture significantly improved clinical effectiveness in both brachial plexus injury (RR = 1.22, 95% CI [1.18, 1.27], *p* < 0.00001) and lumbosacral plexus injury (RR = 1.30, 95% CI [1.19, 1.42], *p* < 0.00001) compared with the non-acupuncture control group (Figure 3A). The test for subgroup differences showed *p* = 0.19 (*p* > 0.05), and the confidence intervals overlapped, indicating that there was no statistically significant difference in the effect of acupuncture between different nerve plexus types, and the results were consistent. Sensitivity analysis confirmed that the results were stable (Figure 3B). In addition, trial sequential analysis (Figure 3C) showed that the cumulative Z-curve crossed the RIS boundary (RIS = 492), indicating that the current data are sufficient to determine that acupuncture is superior to sham acupuncture in improving clinical effective rate.

**Figure 3:**
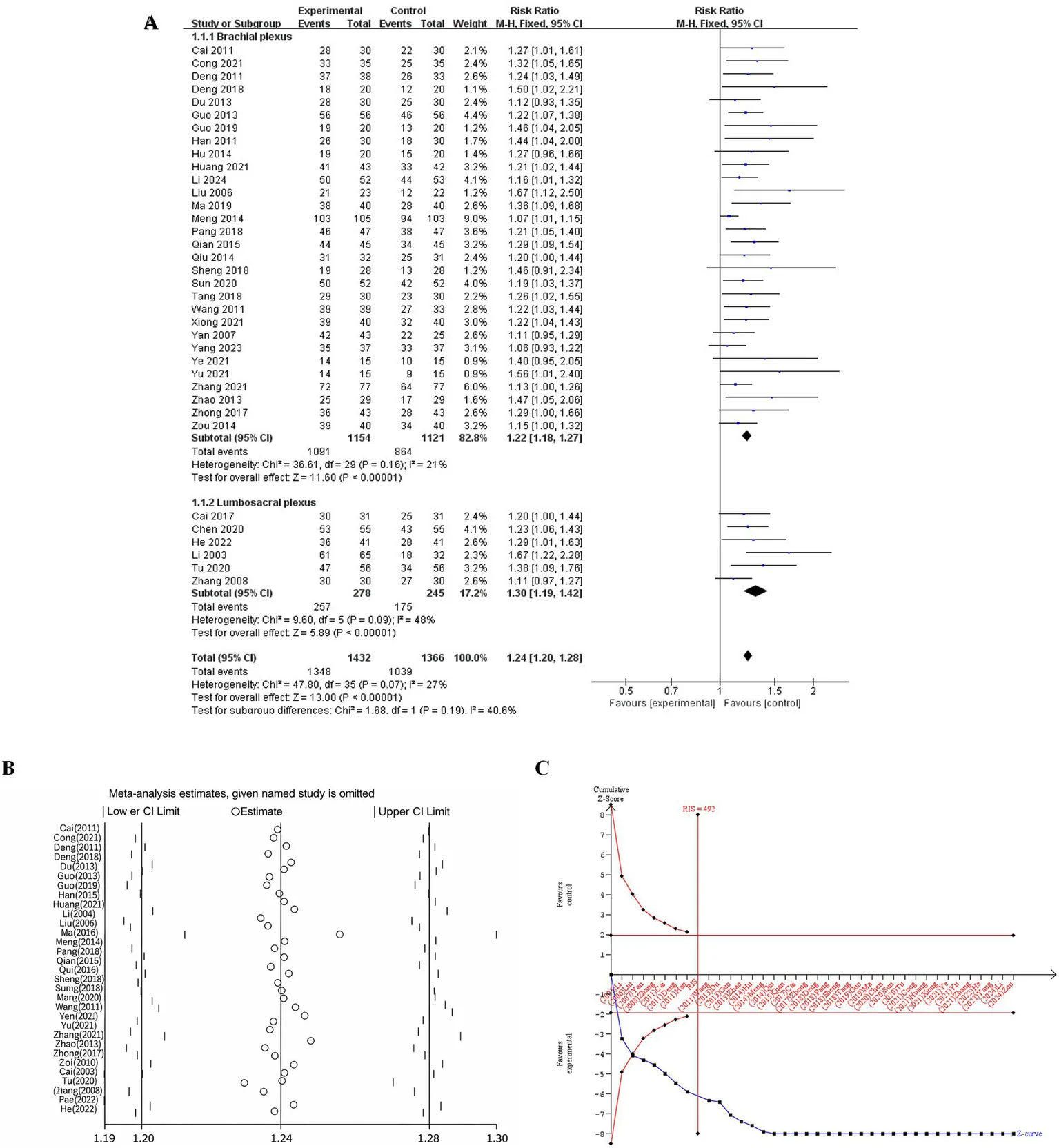
**(A)** Forest plot of clinical effective rate. **(B)** Sensitivity analysis of clinical effective rate. **(C)** Trial sequential analysis of clinical effective rate.

#### 3.3.2 Neurological function score

Five studies (9, 15, 24, 25, 42) reported neurological function scores after treatment in both groups. Among them, two studies (15, 25) were evaluated by physicians based on specific criteria, one study (9) used the Oxford Brachial Plexus Injury Form (OBPIF) scale, and two studies (24, 42) used the Fugl-Meyer Assessment (FMA). Although these scales differ in their item composition and scoring systems, they all share motor, sensory, and reflex functions as their main assessment dimensions. Moreover, previous studies have commonly used them together for outcome evaluation following peripheral nerve injury, suggesting that the three scales are homogeneous in terms of functional assessment. Due to differences in scoring scales, standardized mean difference (SMD) and its 95% CI were used as the pooled effect size. A total of 365 patients were included. Significant heterogeneity was observed (I^2^ = 79%, *p* = 0.0006), and a random-effects model was used. Compared with the non-acupuncture group, acupuncture improved neurological function scores after treatment (SMD = 1.04, 95% CI [0.53, 1.54], *p* < 0.0001; Figure 4A). Subgroup analysis based on nerve plexus showed that acupuncture improved functional recovery in both brachial plexus injury (SMD = 1.11, 95% CI [0.40, 1.81], *p* = 0.002) and lumbosacral plexus injury (SMD = 0.90, 95% CI [0.50, 1.29], *p* < 0.00001) (Figure 4A). Meta-regression analysis showed that injury nerve (*p* = 0.802), intervention duration (*p* = 0.406), intervention frequency (*p* = 0.551), and scoring scale differences (*p* = 0.768) were not sources of heterogeneity. Therefore, the observed heterogeneity could not be fully explained by the included clinical variables. Sensitivity analysis (Figure 4B) confirmed the stability of the results. However, due to differences in item composition and scoring systems across the scales, the pooled results of neurological function scores should be interpreted with caution. TSA (Figure 4C) showed that the cumulative Z-curve crossed the RIS boundary (RIS = 191), indicating that the current data are sufficient to determine that acupuncture is superior to sham acupuncture in improving neurological function scores. However, the results for lumbosacral plexus nerve injury were based solely on a single study (24) and should be considered exploratory findings.

**Figure 4:**
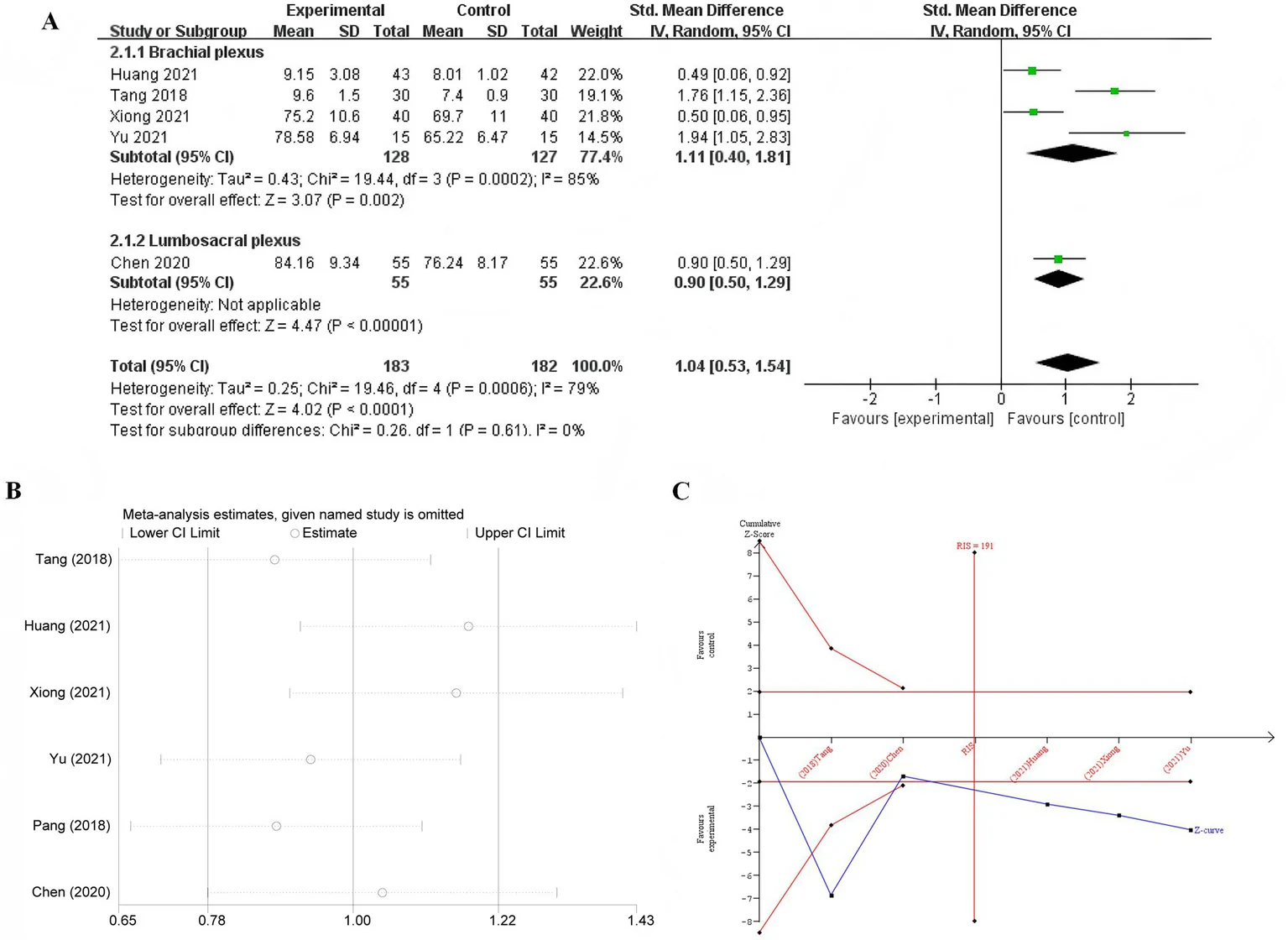
**(A)** Forest plot of neurological function score. **(B)** Sensitivity analysis of neurological function score. **(C)** Trial sequential analysis of neurological function score.

#### 3.3.3 Distal motor latency

Three studies (13, 19, 35) reported DML after treatment, including 226 participants. Significant heterogeneity was observed (I^2^ = 79%, *p* = 0.008), and a random-effects model was used. Compared with the non-acupuncture group, acupuncture reduced DML after treatment (MD = −0.49, 95% CI [−0.78, −0.20], *p* = 0.0008; Figure 5A), indicating that acupuncture can restore nerve conduction function. Subgroup analysis showed that acupuncture improved DML in both brachial plexus injury (MD = −0.64, 95% CI [−0.81, −0.47], *p* < 0.00001) and lumbosacral plexus injury (MD = −0.29, 95% CI [−0.54, −0.04], *p* = 0.02) (Figure 5A). The test for subgroup differences showed (Chi^2^ = 5.40, df = 1, *p* = 0.02, I^2^ = 81.5%), indicating a statistically significant difference between subgroups, with better effects in brachial plexus injury. The difference in the site of nerve injury (brachial plexus vs. lumbosacral plexus) may be a major source of the overall heterogeneity. Meta-regression analysis showed that injury nerve (*p* = 0.670), intervention duration (*p* = 0.602), intervention frequency (*p* = 0.139), and sample size (*p* = 0.460) were not sources of heterogeneity. This suggests that, apart from the site of nerve injury, the other included clinical variables did not explain the remaining heterogeneity, and the heterogeneity may be partly attributable to difficult-to-quantify clinical factors (such as differences in electrophysiological device sensitivity, calibration standards, and operator techniques). Sensitivity analysis (Figure 5B) confirmed stability. TSA (Figure 5C) showed that the cumulative Z-curve crossed the RIS boundary (RIS = 157), indicating sufficient sample size. However, the results for lumbosacral plexus nerve injury were based solely on a single study (13) and should be considered exploratory findings, the stability of which awaits further investigation.

**Figure 5:**
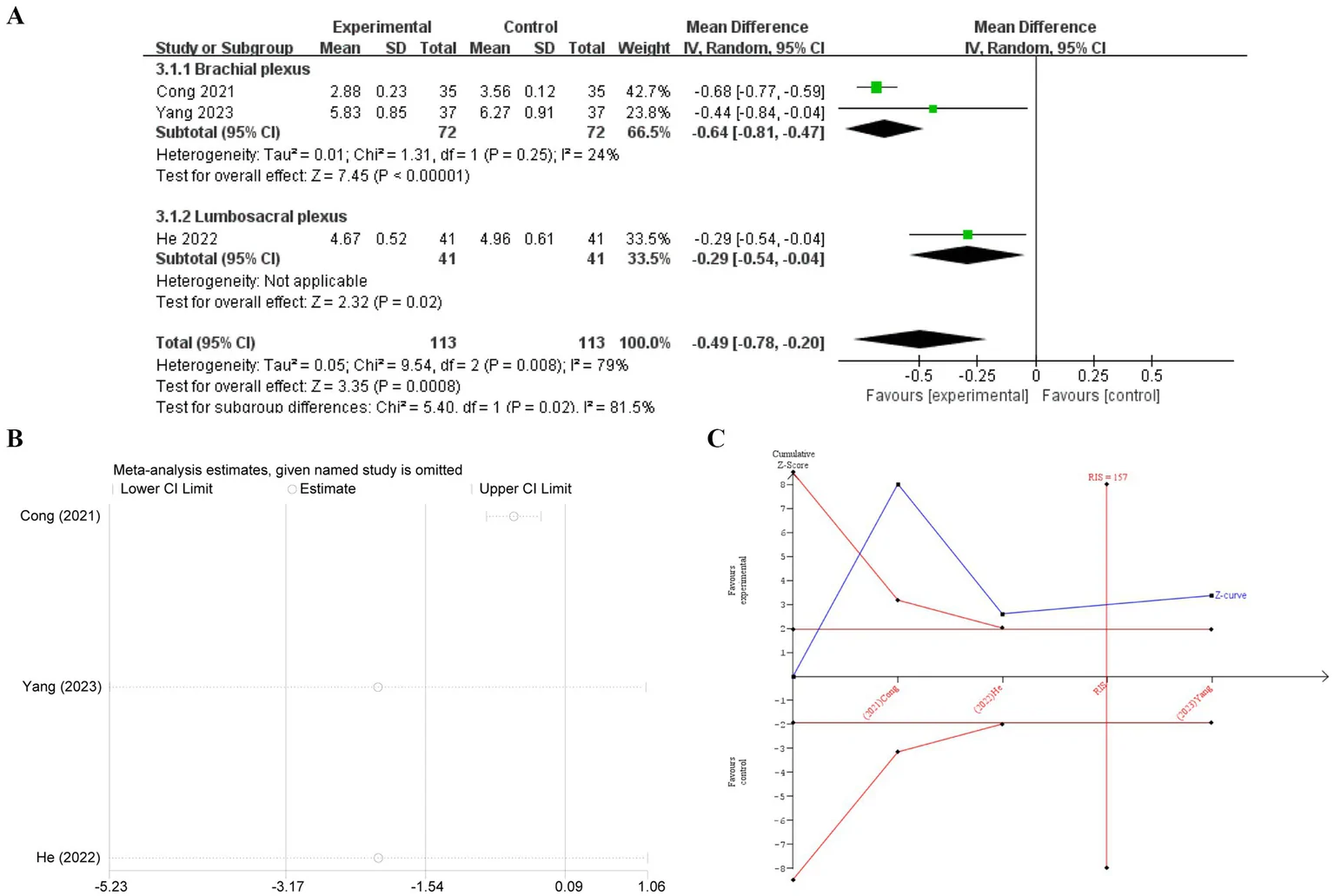
**(A)** Forest plot of distal motor latency. **(B)** Sensitivity analysis of distal motor latency. **(C)** Trial sequential analysis of distal motor latency.

#### 3.3.4 Compound muscle action potential amplitude

Four studies (13, 19, 23, 27) reported compound muscle action potential amplitude (CMAP), including 340 participants. Significant heterogeneity was observed (I^2^ = 98%, *p* < 0.00001), and a random-effects model was used. Acupuncture increased CMAP compared with the non-acupuncture group (MD = 1.88, 95% CI [0.20, 3.56], *p* = 0.03; Figure 6A). Subgroup analysis showed improvements in both brachial plexus injury (MD = 2.45, 95% CI [1.02, 3.88], *p* = 0.0008) and lumbosacral plexus injury (MD = 0.24, 95% CI [0.06, 0.42], *p* = 0.007) (Figure 6A). The test for subgroup differences indicated a significant difference in CMAP improvement between brachial plexus and lumbosacral plexus nerve injuries (Chi^2^ = 9.07, df = 1, *p* = 0.003, I^2^ = 89.0%), with better CMAP recovery in the brachial plexus injury group than in the lumbosacral plexus injury group, also suggesting that the difference in the site of nerve injury is an important source of heterogeneity. Meta-regression analysis showed that acupuncture intervention type (*p* = 0.041) was also a factor influencing the heterogeneity. Sensitivity analysis (Figure 6B) confirmed stability. TSA (Figure 6C) showed that the cumulative Z-curve did not cross the RIS boundary (RIS = 649), suggesting that this finding requires further verification in larger-sample clinical trials. Meanwhile, the results for lumbosacral plexus nerve injury were based solely on a single study (13) and should be regarded as exploratory findings rather than confirmatory conclusions.

**Figure 6:**
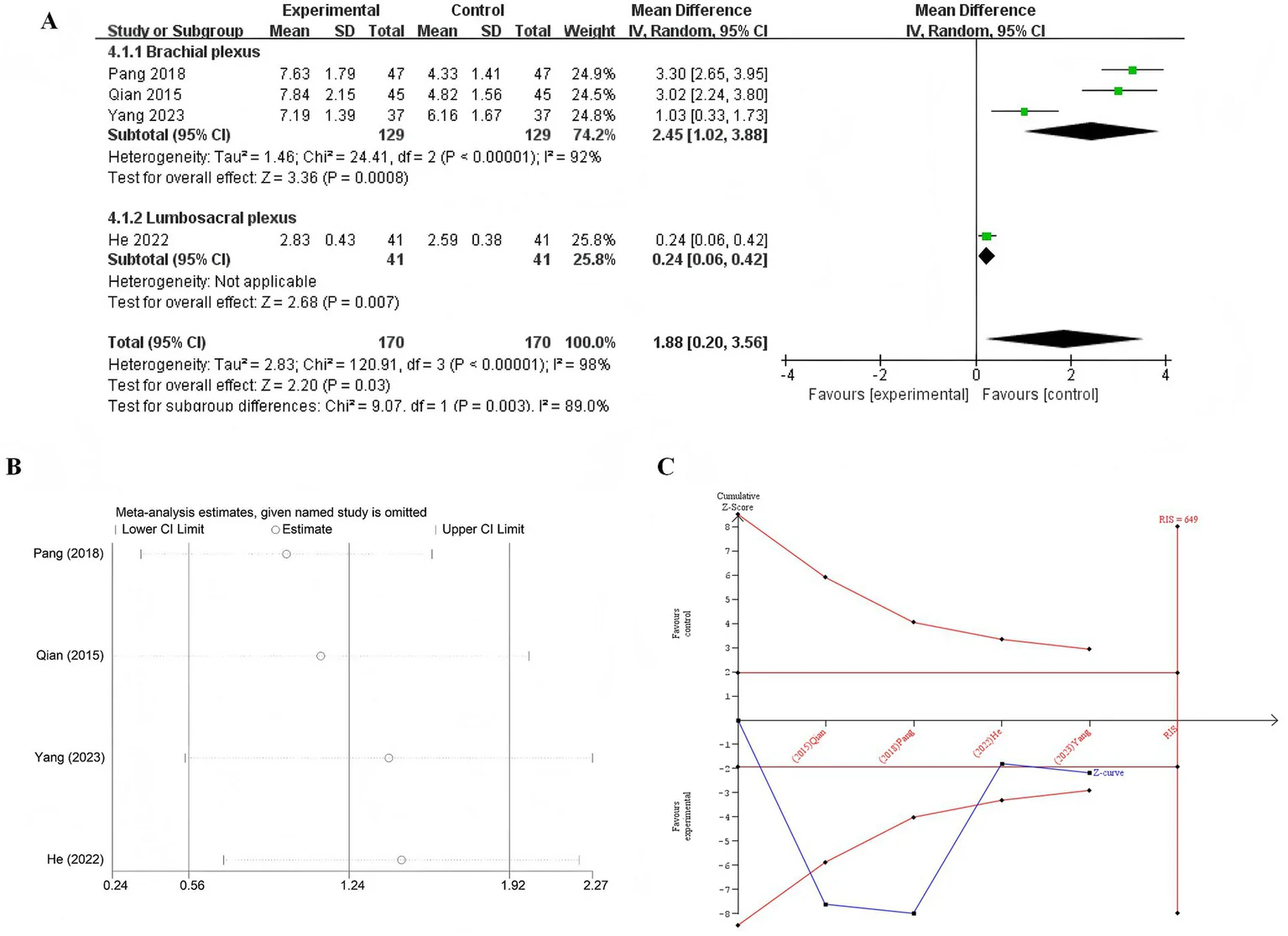
**(A)** Forest plot of compound muscle action potential amplitude. **(B)** Sensitivity analysis of compound muscle action potential amplitude. **(C)** Trial sequential analysis of compound muscle action potential amplitude.

#### 3.3.5 Sensory nerve conduction velocity

Five studies (10, 13, 19, 35, 40) reported sensory nerve conduction velocity (SCV), including 410 participants. Significant heterogeneity was observed (I^2^ = 81%, *p* = 0.0004), and a random-effects model was used. Acupuncture increased SCV compared with the non-acupuncture group (MD = 5.14, 95% CI [2.33, 7.95], *p* = 0.003; Figure 7A). Subgroup analysis showed improvement in brachial plexus injury (MD = 6.15, 95% CI [3.31, 8.98], *p* < 0.0001), while no significant difference was observed in lumbosacral plexus injury (MD = 1.58, 95% CI [−0.24, 3.40], *p* = 0.09) (Figure 7A). The test for subgroup differences showed a significant difference between the two groups (Chi^2^ = 7.05, df = 1, *p* = 0.008, I^2^ = 85.8%), suggesting that there is a substantial difference in the effect of acupuncture on SCV improvement between brachial plexus and lumbosacral plexus nerve injuries, and this clinical factor may be the primary source of the overall heterogeneity. Meta-regression analysis further showed that injury nerve (*p* = 0.397), intervention duration (*p* = 0.453), intervention frequency (*p* = 0.409), disease duration (*p* = 0.393), and sample size (*p* = 0.691) were not sources of heterogeneity, suggesting that, apart from the site of nerve injury (brachial plexus vs. lumbosacral plexus), none of the other included clinical variables could explain the remaining heterogeneity. Sensitivity analysis (Figure 7B) confirmed stability. TSA (Figure 7C) showed that the cumulative Z-curve crossed the RIS boundary (RIS = 250), indicating sufficient sample size. It is worth noting that the results for lumbosacral plexus nerve injury were based solely on a single study (13) and should be regarded as exploratory analyses, the stability of which requires further investigation.

**Figure 7:**
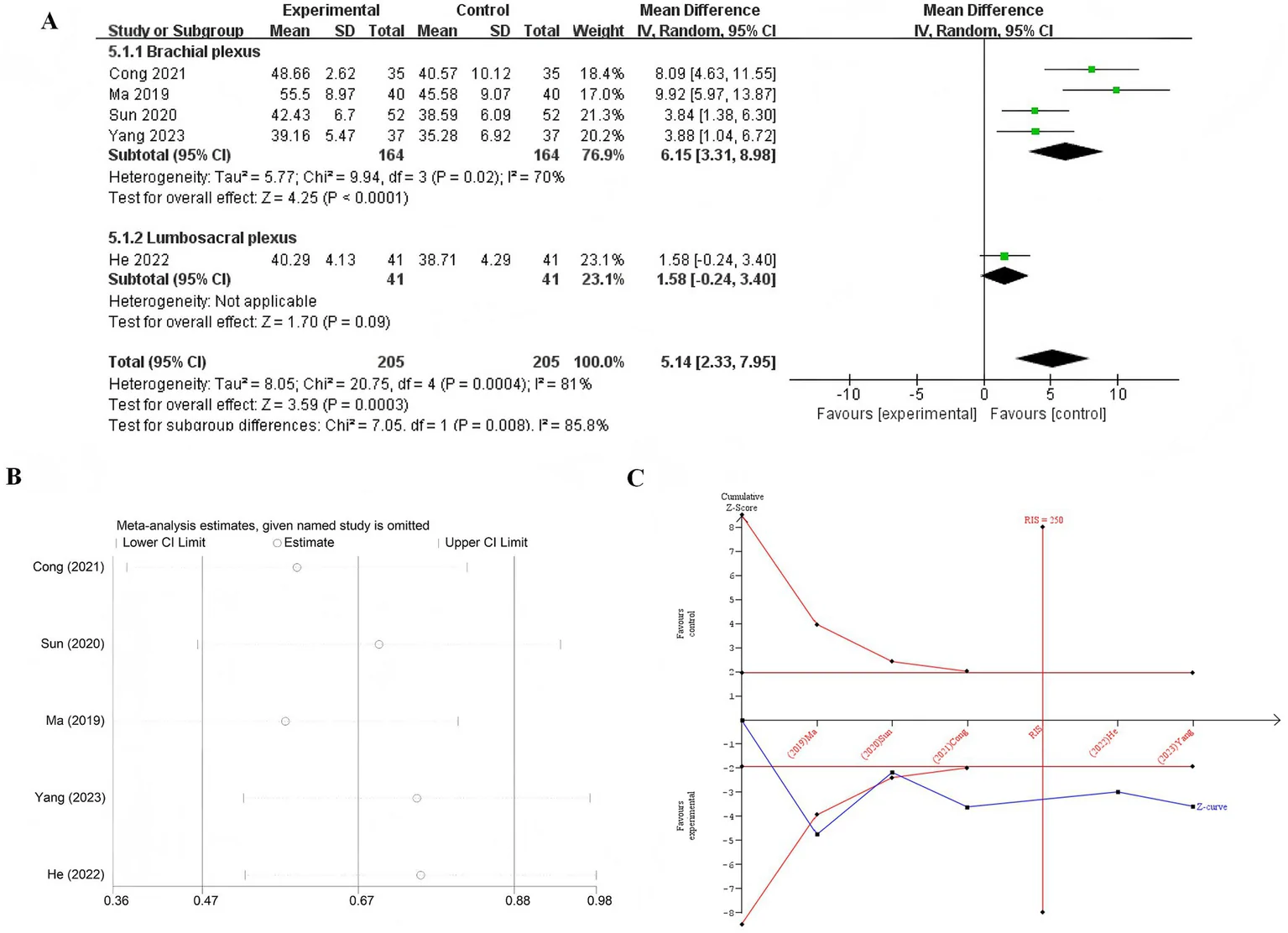
**(A)** Forest plot of sensory nerve conduction velocity. **(B)** Sensitivity analysis of sensory nerve conduction velocity. **(C)** Trial sequential analysis of sensory nerve conduction velocity.

### 3.4 Adverse events

No serious adverse events related to acupuncture were reported in the included studies.

### 3.5 Publication bias

A total of 36 studies reporting clinical effectiveness were included for publication bias analysis. A funnel plot was generated using RevMan software (Figure 8), showing some asymmetry in the distribution of effect sizes. However, Egger’s test did not detect significant publication bias (*p* = 0.892). The trim-and-fill method added 10 studies, improving the symmetry of the funnel plot without changing the results, indicating that there was no publication bias and that the results were stable and reliable.

**Figure 8:**
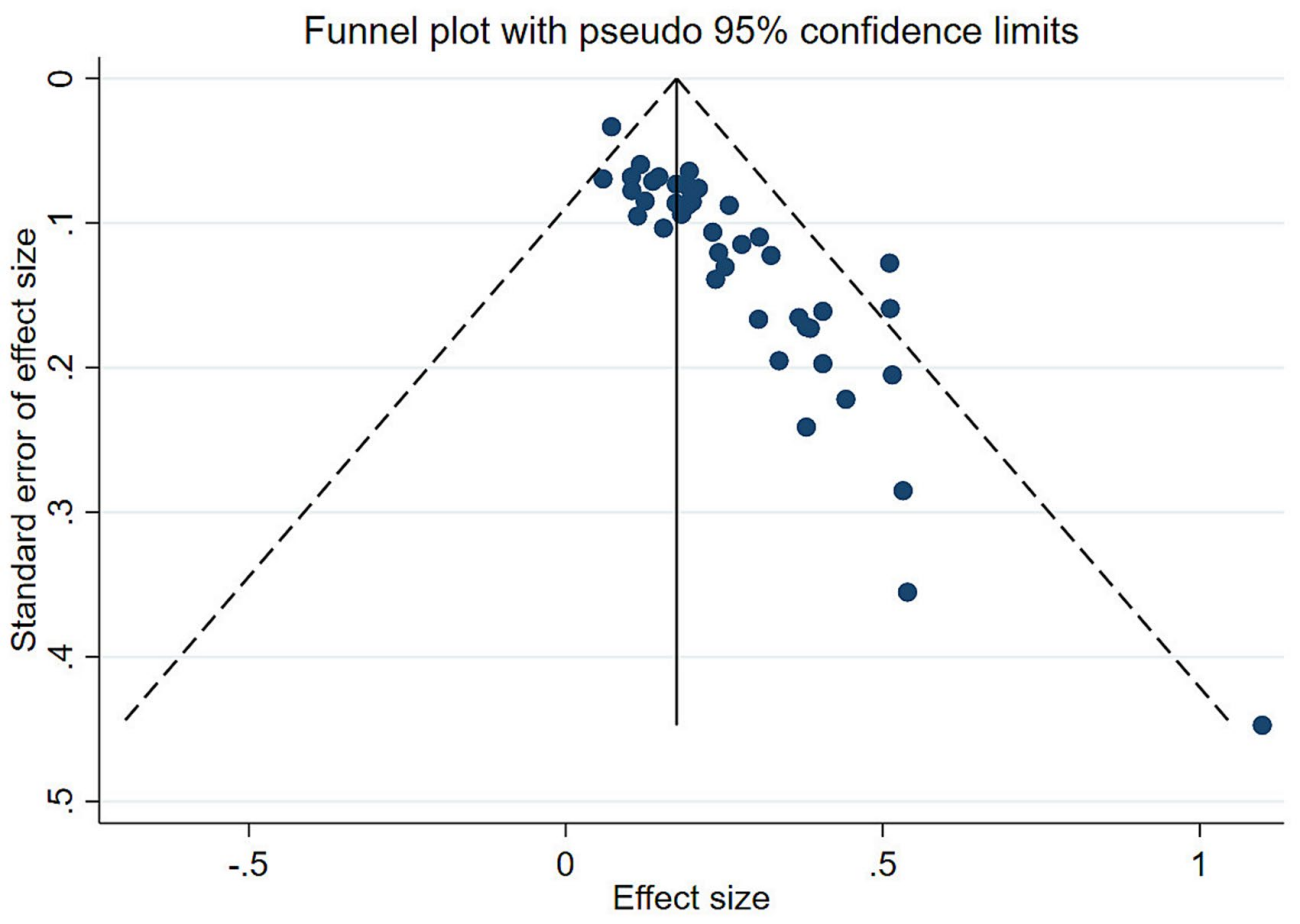
Funnel plot of publication bias.

### 3.6 Certainty of evidence

Overall, the certainty of evidence was rated as low to very-low for electrophysiological outcomes including neurological function score, DML, CMAP and SCV, while clinical effective rate presented moderate-certainty evidence. The downgrading of evidence certainty may be mainly attributed to the risk of methodological bias, substantial heterogeneity across studies and relatively small sample sizes of the included trials. Supplementary Table 1 presents the results of the GRADE analysis.

## 4 Discussion

The results showed that acupuncture is a feasible therapy for traumatic peripheral nerve injury (TPNI), and no serious adverse events were reported. Compared with the non-acupuncture control group, acupuncture can improve the clinical efficacy of TPNI and enhance patients’ motor control ability, sensory function, and quality of life. Previous meta-analyses have shown that acupuncture is superior to non-acupuncture treatment in patients with brachial plexus injury in terms of overall effective rate, electromyography outcomes, and brachial plexus function scores (45), but these studies did not systematically and comprehensively analyze the overall efficacy of acupuncture for TPNI, nor did they explore whether the included sample size was sufficient to draw stable conclusions. Therefore, in this study, while evaluating the clinical efficacy of acupuncture for patients with TPNI, trial sequential analysis was used to assess whether the sample size was sufficient to obtain stable conclusions.

TPNI can lead to the loss of motor, sensory, and autonomic nerve functions, and the recovery process is highly complex and uncertain (46). A single indicator is difficult to fully reflect the overall recovery, so it is necessary to combine subjective perception, objective function, and electrophysiological activity for multidimensional analysis (47). In this study, 36 studies meeting the inclusion and exclusion criteria were analyzed qualitatively and quantitatively. In terms of clinical effective rate, neurological function score, DML, and SCV, the acupuncture group showed statistically significant differences compared with the non-acupuncture control group, and trial sequential analysis confirmed that the included sample size was sufficient to obtain stable results. For CMAP, acupuncture showed a statistically significant difference compared with sham acupuncture, but due to insufficient sample size, stable conclusions could not be drawn, and further clinical trials with larger sample sizes are needed.

Subgroup analysis based on nerve plexus showed that acupuncture improved clinical effective rate and neurological function score in patients with different nerve plexus injuries, and there was no statistically significant difference between subgroups, indicating that acupuncture is effective for both brachial plexus injury and lumbosacral plexus injury, with similar efficacy. For DML and CMAP, acupuncture improved outcomes in different nerve plexus injuries, and the effect on brachial plexus injury was better than that on lumbosacral plexus injury, with statistically significant differences between subgroups. For sensory nerve conduction velocity, significant improvement was observed only in the brachial plexus subgroup, while acupuncture showed a favorable but non-significant trend toward improving SCV in lumbosacral plexus injury. Additionally, among the secondary outcome measures, including neurological function scores, DML, CMAP, and SCV, the effect estimates for lumbosacral plexus nerve injury were based solely on a single trial and should be considered exploratory analyses in nature, the stability of which requires further investigation.

In this study, most outcome measures exhibited substantial statistical heterogeneity. Subgroup analyses suggested that the difference in injury site between the brachial plexus and lumbosacral plexus may be a major source of heterogeneity for SCV, CMAP, and DML, while the source of heterogeneity for neurological function scores may be related to other difficult-to-quantify factors. Furthermore, the control interventions in the included studies varied, including conventional Western medication, physical rehabilitation training, and biofeedback therapy, which were uniformly pooled into a non-acupuncture control group in this study, potentially introducing additional clinical heterogeneity and thereby contributing to the elevated I^2^ values.

In terms of evidence quality, this study further employed the GRADE system to assess the certainty of the pooled effect estimates. This approach integrates five domains—risk of bias, inconsistency, indirectness, imprecision, and publication bias—to evaluate the certainty of evidence for five outcome measures derived from 36 studies. The results showed that the clinical effectiveness rate was rated as moderate-certainty evidence; DML as low-certainty evidence; and the three electrophysiological outcomes, including neurological function scores, CMAP, and SCV, as very low-certainty evidence. No outcome was rated as high certainty, indicating an overall low level of evidence certainty. The main reasons for downgrading the GRADE ratings included: (1) most of the included randomized controlled trials did not adequately report allocation concealment methods nor implement blinding of participants and personnel. It should be noted that, due to the specific nature of acupuncture procedures, double-blinding of acupuncturists and participants is not feasible; (2) substantial statistical heterogeneity was observed in some outcome measures, and the sources of heterogeneity may be related to differences in acupuncture sites and intervention modalities; (3) TSA results showed that although the Z-curves for CMAP and SCV crossed the conventional statistical significance boundary, the cumulative sample sizes had not yet reached the required information size; whereas the Z-curve for neurological function scores did not cross the conventional significance boundary, suggesting that the current cumulative evidence remains insufficient and the stability of the evidence is inadequate. Therefore, this indicates that more high-quality randomized controlled trialsare needed to further verify the effectiveness of acupuncture in treating TPNI.

## 5 Strengths and limitations

This study conducted a multidimensional systematic evaluation of acupuncture in the treatment of TPNI, and subgroup analysis based on nerve plexus was performed to explore the efficacy of acupuncture. Additionally, trial sequential analysis was used to evaluate whether the included sample size was sufficient to support stable conclusions, which provides a reference for the design of future clinical trials on acupuncture for peripheral nerve injury.

The limitations of this study are as follows. It should be noted that only studies published in Chinese and English were included, and relevant studies published in other languages may have been missed, resulting in potential language bias. Additionally, all included studies were conducted in China and all reported positive results. The geographical limitation of the sample sources precludes determination of whether the findings are generalizable to populations in other countries. This phenomenon is consistent with the previously reported “Chinese acupuncture literature effect,” in which the proportion of positive results in acupuncture clinical trials for neurological diseases from China is higher than that in other countries and regions (48). The possible reasons include: a publication preference for positive findings among Chinese domestic journals, the risk of non-publication of negative or neutral results, and methodological flaws in some studies that may lead to false-positive findings. Therefore, the pooled effect sizes in this study can be potentially overestimated. In the future, efforts should be made to further encourage the publication of methodologically sound negative trials and to promote transparency in clinical trial results and study protocols, so as to reduce the impact of publication bias on pooled effect sizes.

## 6 Conclusion

As an adjunctive therapy, acupuncture has advantages in improving the rate of neurological function recovery in patients with traumatic peripheral nerve injury and is associated with no adverse reactions. However, given the suboptimal methodological quality of the included reviews, the current evidence does not yet permit definitive conclusions. Future well-designed, high-quality randomized controlled trials are needed to further enhance the certainty of our findings.

## Data Availability

The original contributions presented in the study are included in the article/Supplementary material, further inquiries can be directed to the corresponding authors.

## Supplementary material

SUPPLEMENTARY TABLE S1 GRADE certainty‑of‑evidence assessment.
