# Supplementary material for: Clinical effects of acupuncture for traumatic peripheral nerve injury: a meta-analysis of randomized controlled trials
Source: Front Neurol. 2026 Oct 16;17:1852669. doi: 10.3389/fneur.2026.1852669 (PMC13720883; doi:10.3389/fneur.2026.1852669)
Supplement: Supplementary file 1 [file Supplementary_file_1.docx]

| **Certainty assessment** | | | | | | | **№ of patients** | | **Effect** | | **Certainty** | **Importance** |
| --- | --- | --- | --- | --- | --- | --- | --- | --- | --- | --- | --- | --- |
| **№ of studies** | **Study design** | **Risk of bias** | **Inconsistency** | **Indirectness** | **Imprecision** | **Other considerations** | **experimental group** | **control group** | **Relative (95% CI)** | **Absolute (95% CI)** |  |  |
| **Clinical effective rate** | | | | | | | | | | | | |
| 36 | randomised trials | serious^a^ | not serious | not serious | not serious | none | 1348/1432 (94.1%) | 1039/1366 (76.1%) | **RR 1.24** (1.20 to 1.28) | **183 more per 1,000** (from 152 more to 213 more) | ⨁⨁⨁◯ Moderate^a^ |  |
| **Neurological function score** | | | | | | | | | | | | |
| 5 | randomised trials | serious^a^ | serious^b^ | not serious | serious^c^ | none | 183 | 182 | - | SMD **1.04 higher** (0.53 higher to 1.54 higher) | ⨁◯◯◯ Very low^a,b,c^ |  |
| **DML** | | | | | | | | | | | | |
| 3 | randomised trials | serious^a^ | serious^b^ | not serious | not serious | none | 113 | 113 | - | MD **0.49 lower** (0.78 lower to 0.2 lower) | ⨁⨁◯◯ Low^a,b^ |  |
| **CMAP** | | | | | | | | | | | | |
| 4 | randomised trials | serious^a^ | serious^b^ | not serious | serious^d^ | none | 170 | 170 | - | MD **1.88 higher** (0.2 higher to 3.56 higher) | ⨁◯◯◯ Very low^a,b,d^ |  |
| **SCV** | | | | | | | | | | | | |
| 5 | randomised trials | serious^a^ | serious^b^ | not serious | serious^d^ | none | 205 | 205 | - | MD **5.14 higher** (2.33 higher to 7.95 higher) | ⨁◯◯◯ Very low^a,b,d^ |  |

**CI:** confidence interval; **MD:** mean difference; **RR:** risk ratio; **SMD:** standardised mean difference.

**Explanations** : ^a^The included studies had methodological limitations (e.g., lack of allocation concealment and blinding). ^b^Substantial heterogeneity was observed. ^c^Despite reaching the RIS, the Z-curve in TSA did not cross the significance boundary, suggesting limited robustness. ^d^The Z-curve crossed the significance boundary, but the sample size did not reach the RIS, suggesting imprecision.
